# Molecular recognition at interfaces. Adhesion, wetting and bond scrambling

**DOI:** 10.3389/fchem.2022.1088613

**Published:** 2022-12-09

**Authors:** Alberto Ciferri

**Affiliations:** Chemistry Department, Duke University, Durham, NC, United States

**Keywords:** cohesive energy, surface energy, solubility parameters, binding constants, adhesion, wetting, molecular recognition, bond scrambling

## Abstract

The quantitative description of the supramolecular interaction occurring at the adhesion surfaces of different polymers has enabled elaborate dissections of contributions to cohesive and surface energies. An alternative analysis is proposed here based on solubility parameters and binding constants that traditionally describe the weakest and relatively larger association energies in polymer blends. The article emphasizes a feature of supramolecular polymers that has not received adequate consideration: The dynamic bond scrambling that allows a most efficient molecular recognition over significant areas of synthetic and biological surfaces.

## Introduction

Adhesion is the ability of two phases, usually two solid surfaces of similar or dissimilar substances, to bind (cling together) by virtue of supramolecular interaction or mechanical interlocking. Wetting is the term used in the case of a solid-liquid interface Awaja et al., (2009). Typical practical examples involving polymers include coating and painting (Zheng, 2020), wetting as in the printing ink industry (Cern et al., 2021), cases in which adhesion is enhanced by promoters (i.e., chemicals that act at the interface between an organic polymer and an inorganic substrate) (Peter, 2011). Relevant are also cases of self-healing (when two fractured surfaces of the same polymer will seal again if brought in close contact_)_ (Ciferri, 2013)_._ Among biological materials, the enzyme-substrate complex shows a remarkable association mechanism, extending to the most complex cases of cell adhesion associated with a variety of function (Buckley et al., 1998; Ciferri, 2021).

There is not a general molecular theory for chemical adhesion. However, several studies have highlighted the need of dissecting the supramolecular components of cohesive and surface energies that control solubility, surface wettability and the strength of the interfacial association (Hansen, 2007; Abdullah et al., 2015; Nanoscience Instruments; Ciferri, 2021). The present note includes a review of cohesive and surface energies and suggests the application of the classical parameters that control the interactions of supramolecular polymers, emphasizing their bond scrambling features.

## Cohesive energy

The cohesive energy (*ω*_*coh*_) of a substance in the bulk phase is defined by the internal energy per mole that results from the balance of all its supramolecular interactions (Flory, 1953; Hildebrand et al., 1970; Burke, 1984). Cohesive energy densities can be rigorously determined using *ab initio* calculations (Abdullah et al., 2015). For low molecular weight substances, the cohesive energy is the energy required to evaporate the material, assessed from the heat of vaporization in calories per cubic centimeter. For materials that decompose before evaporating, cohesive energies are often evaluated from known group contributions (Marsano et al., 1984).

Cohesive energy is also related to solubility parameters. The Hildebrand solubility parameter (*δ*) is defined by the square root of the cohesive energy density (Hildebrand et al., 1970):

$$\delta ={\left(\Delta H/V\right)}^{1/2}$$ (1)

where _*Δ*_H is the measurable enthalpy of vaporization and V the molar volume. The Hildebrand solubility parameter is related to the Flory-Huggins solubility parameter χ (Flory, 1953):

$$\chi_{12}=z\;\Delta \omega /kT$$ (2)

where z is the coordination number and Δω expresses the energy change upon rupture of contacts between each of the two components of a mixture with formation of new contacts between these components (Flory, 1953):

$$∆\omega =\omega_{12}-1/2\left(\omega_{11}+\omega_{22}\right)$$ (3)

The concept of solubility is relevant to the description of cohesive energy since it involves the rupture and the association of two compounds. The relationship between the Hildebrand and the Flory-Huggins solubility parameters is:

$$\chi_{12}=V_{1}/RT\;{\left(\delta_{1}-\delta_{2}\right)}^{2}$$ (4)

where $\delta_{1}$ and $\delta_{2}$ are the solubility parameters of solvent and polymer, and V_1_ is the molar volume of the solvent (Hildebrand et al., 1970).

The Flory-Huggins free energy of mixing real solutions (Flory, 1953) includes different contributions, in particular one expressing the ideal mixing entropy, another expressing weak interactions through the diluent parameter χ (Flory, 1953), and another expressing stronger attractive interactions through binding constant k_j_ (Orofino et al., 1967). A relevant expression for the melting temperature depression of a polymer in a binary diluent is (Orofino et al., 1967):

$$1/T_{m}-1/T_{m}^{0}=\left(R/\Delta Η\right)\left(v_{1-}\;x_{12}\;v_{1}^{2}\right)+\left(R/\Delta Η\right)\;\mathrm{pln}\left(1+K_{j}c_{j}\right)$$ (5)

On the right-hand side (RHS), χ_12_ and K_j_ equal zero for an ideal solution, p is the number of binding sites and c_j_ represents the activity of a binding agent. Different solvents and different binding agents (i.e., water, salts….) can be evaluated with the latter equation. Corresponding Kj could be evaluated from enrichment data (Orofino et al., 1967). We discuss separately the cases of weak and relatively stronger interactions.

### Weak supramolecular interactions

In terms of current polymer solution theory (Flory, 1953) any weak favorable attraction between the components (good solvents) is characterized by negative values of χ_12_, whereas repulsive interactions, including excluded volume effects, are characterized by positive values of χ_12._

The work of Blanks and Prausnitz stands above the earliest investigations of polymer solutions (Blanks and Prausnitz, 1984). They demonstrated that the validity or self-consistency of the above relationships is restricted to systems with low polarity, primarily due to van der Waals interactions.

They analyzed different solvents and model substances that included three components, namely dispersive *(*London) interactions (induced by transient dipoles even in non-polar molecules), polar (Debye) interactions between permanent dipoles, and Keeson interactions between permanent and induced dipoles (Hiemenz and Rajagopalan, 1997). Table 1 reveals that the global van der Waals interactions are the weakest supramolecular interactions, having association strength below 1 kcal/mol and active separation distances smaller than about 0.5 nm. Corresponding χ_12_ values evaluated by Blanks and Prausnitz are in the range −0.1 to + 0.5^16^.

**TABLE 1:** Cohesive supramolecular energies.

Interaction	Strength (kj/mol)	Distance (nm)
Dispersive or van der Waals^a^	0.4–4.0	0.3–0.6
Hydrog. bond	12–30	0.3
Ionic	20	0.25
Hydrophobic	<40	varies

^a^ Include London, Keeson, Debye forces.

Table 2 evidences a more detailed evaluation of the separate contributions to van der Waals interaction. The results are based on a sophisticated approach, primarily the temperature and enthalpies of evaporation (Hiemenz and Rajagopalan, 1997). Included are representative data for molecules chosen as to have comparable values of dipolar moment and polarizability (the latter is a measure of the easiness of electrons to move around the molecule in response to an external electric field or a neighboring dipole) (Hiemenz and Rajagopalan, 1997). The data show that dispersive London interactions are generally prevailing. Nevertheless, the dipolar moment and the molecular polarizability also have a role, complicating the assignment of the various contributions. Additional difficulties have been more recently reported, suggesting that solvents may mitigate the intensity of the components evaluated from vaporization enthalpies (Young et al., 2013). In fact, cohesive solvent-solvent interactions (*cf.*, Eq. 3) were found to be the major driving force for apolar association in solution (Young et al., 2013).

**TABLE 2:** Percentage of the Debye (permanent-permanent), Keeson (permanent-induced) and London (induced-induced) contributions to van der Waals dipolar interactions, as related to the permanent dipole and polarizability of typical compounds.

Compound	Perm. Dipole^a^	Polarizability^b^	% debye	%Keeson	% london
Ccl4	100.0	0.00	10.70	0.0	0.0
Benzene	100.0	0.00	10.50	0.0	0.0
Toluene	99.0	0.43	11.80	0.1	0.9
Aniline	77.9	1.56	12.40	13.6	8.5
Ethanol	47.6	1.73	5.42	42.6	9.7
Water	10.5	1.82	1.44	84.8	4.5

Data from Table 10.2 of reference 17.

^a^ D units.

^b^ C m^−2^ units.

### Stronger supramolecular interactions

Stronger specific interactions occurring between polymeric solutes may be described by a binding constant (Orofino et al., 1967). An example is the electrostatic attraction between a proton in one molecule and an electronegative atom in another one forming the intermolecular hydrogen bond having dissociation constants in the range 10–30 kcal/mol (Steiner, 2002), see Table 1. Other strong interactions include ϖ-ϖ (Ma et al., 2021), hydrophobic and ion binding interactions (see Table 1). Ion binding typical of salting-in ions in the Hofmeister series (i.e., calcium ions) can also induce intra or intermolecular bridges (Ciferri et al., 2012).

An approach allowing a quantitative assessment of weak van der Waals and stronger bonding contributions is afforded by the generalized Flory-Huggins Eq. 5. The χ_12_ parameters and equilibrium constants for ionic interactions were evaluated from viscosity analysis and enrichment data for gelatin-water solutions in the presence of salts of the Hofmeister series (Ciferri et al., 2012). The analysis provided definite evidence that the solubility of proteins is controlled by van der Waals interactions in the case of typical salting-out agents (i.e., KCl) and by specific binding in the case of typical salting-in agents (i.e., CaCl_2_) (Ciferri et al., 2012). No values for K_w_ relevant to H-bonding were reported.

An alternative thermodynamic approach to describe the simultaneous occurrence of weak and specific supramolecular interactions was elaborated by Hanson (Hansen, 2007). He suggested that the global interaction could be dissected into three additive components characterizing, respectively, the dispersive *(*London), polar (Debye) and hydrogen bonding contributions.

$$\sigma =\sigma_{d}+\sigma_{p}+\sigma_{h}$$ (6)

Various analytical approaches were elaborated to dissect the three components from raw solubility data. A complex approach based on inverse gas chromatography is detailed by Adamska and Voelkel (2006). The solubility behavior relevant to a variety of technological processes was reasonably well described by the Hansen parameters (Hansen, 2007). These parameters were also used to assess the compatibility between a dispersed and a continuous phase (Bapat et al., 2021).

Specific selections of the relevant components of the cohesive energy may be relevant to special formulations for industry (Abdullah et al., 2015; Haixia et al., 2021).

## Surface energy

Within a bulk phase, the interaction forces on an atom/molecule are mutually equilibrated whereas unbalanced interactions prevail at the surface. Such an unbalanced energy is referred to as the surface “energy” in the case of attractive interactions between two solid substances. Surface “tension” is instead when liquids are involved. The surface energy/tension increases with number of bulk interactions. A high surface energy refers to a strong molecular attraction, whereas a low surface energy refers to a weak molecular attraction or low compatibility (cph Deutschland Chemie GmbH, 2020; Brighton Science, 2021).

Whereas adhesion is the term preferentially uses for association of two solid surfaces, wetting is used when a liquid spreads over a solid. In contrast to wetting, an incompatible liquid will form a drop characterized by a contact angle. The boundary between solid-solid or solid-liquid systems is referred to as the interface (cph Deutschland Chemie GmbH, 2020; Brighton Science, 2021).

Surface energy is often assessed using contact angle data obtained for solid-liquid systems. The Yung equation allows a quantitative relationship between the surface energies of solid and liquids (*σ*_*s*_ and *σ*_*l*_), the interfacial tension between liquid and solid $\left(\sigma_{sl\;}\right)$ and the angle (*θ*) between the liquid on the solid (Data Physics Instruments):

$$\sigma_{s}=\sigma_{sl}+\sigma_{l}\;\cos Ɵ$$ (7)

Several methods for the evaluation of the surface tension of solids and liquids are described in the literature. Contact angles of test liquids may be used (Cheng et al., 1990). In the case of incompatible liquids, determination of contact angles with an optical goniometer may be facilitated by drop shape analysis (Bouge et al., 1986).

There is some evidence that surface energy is affected by chain conformation (Flory, Statistical, 1969; Chalykh, 2020; Dataphysics, 2021). The bulk conformation of flexible polymers is often described as that prevailing under theta conditions when excluded volume effects are balanced by supramolecular attraction. More rigid polymers are characterized by parallel orientation of the macromolecular chains in the surface layer (Dataphysics, 2021). The latter effect has recently been shown to correspond to an enhancement of surface energy.

A model for the adhesion mechanism proposed by Data Physics (Dataphysics) is based on only two types of surface energy, one involving transient dipoles (included in dispersive interactions, *cf.* Table 1) and the other polar (Debye and H-bond) interactions.

$$\sigma =\sigma^{d}+\sigma^{p}$$ (8)

**FIGURE 1:**
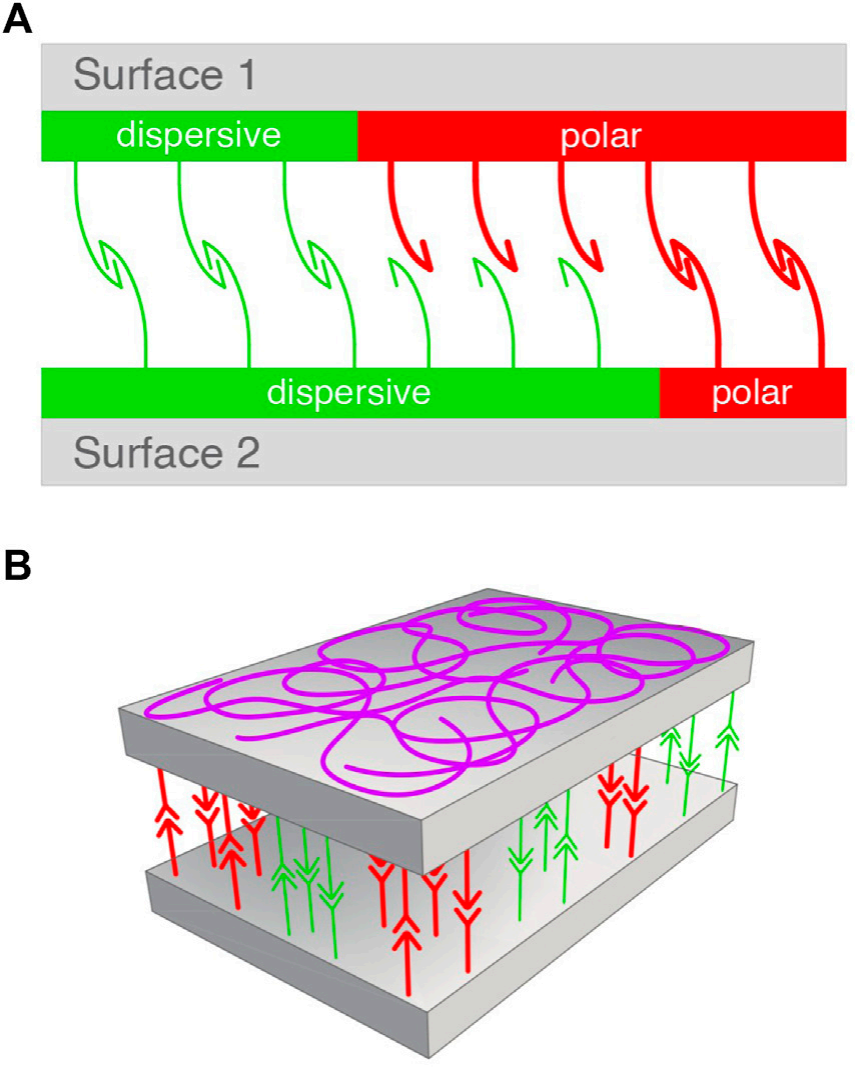
Models for surface association. **(A)** Data-Physics model based on dispersive (green) and polar interactions (red) balanced between two surfaces (Eq. 8). Adapted from Dataphysics. **(B)** Presently suggested model based on balanced and randomly oriented weak interactions represented by the Flory’Huggins parameter (green), and a stromger binding constant (red) (Eq. 5). All supramolecular bonds undergo dynamic scrambling (exchanging partners) under equilibrium conditions. *Designer: Luca Galbusera, 2022.*

These interactions were schematized on a simple figure in which the adhesion of two surfaces was promoted by the recognition of the two types of bonds (Figure 1A) (Dataphysics). The model based on our Eq. 5 is also based on just two types of interactions: one based on the χ_12_ parameter (representing all weak interactions of the van der Waals type, the other all strong interactions described by an equilibrium constant K). Thus, we include in Figure 1B a schematization of how two surfaces should bind, consistently with general principles of supramolecular polymers (Ciferri, 2016; Ciferri, 2020).

## Bond scrambling

Bond scrambling is a fundamental motion occurring in processes such as reversible chemical reactions and supramlecular associations, both characterized by small equilibrium constants. In these processes, an active terminal group continues to associate and dissociate various complementary groups. The motion greatly assists the recognition of binding partners even in the absence of mechanical sliding or main chains reorganization. A dynamic equilibrium will be reached when the rates of association and dissociation with adjacent oligomers are equal. Under equilibrium, alteration of the composition of the reactants (i.e., the degree of polymerization, DP) is not allowed and a direct relationship between DP and the binding constant is predicted and experimentally verified (i.e., DP ∼ K^1/2^) (Ciferri, 2016). Nevertheless, bond scrambling motion can affect some labile structurization.

**FIGURE 2:**
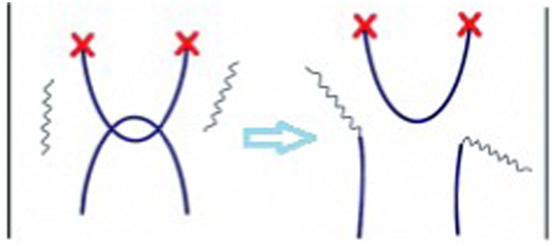
Breaking e chain entanglements relieves steric constraints.

As first suggested by J. M. Lehn, bond scrambling allows the elimination of steric constraints in supramolecular networks (i.e., entanglements, see schematization in Figure 2) (CiferriBond and Network Elasticity, 2009). These networks have indeed been described as dynamic materials having adaptive features (Cordier et al., 2008). Similar features have been documented even for hydrogels (Sinawang et al., 2020). Moreover, scrambling has also been shown to induce the randomization of ordered sequences (Bleiholder et al., 2008). The role of scrambling in promoting a chain size distributions has also been documented (Matula et al., 1964; Moedritzer, 1964).

Particularly relevant to surface recognition is the self-healing of freshly fractured surfaces of a given polymer (Cordier et al., 2008; CiferriBond and Network Elasticity, 2009)^.^ In this case, it could be argued that the detailed location and orientation of proton donors and acceptors of the H-bonds, severed during fracture, might have been only slightly altered, thus aiding the recognition process.

A more complex mismatch between the position and orientation of the H-bond components is instead expected when two different surfaces of a non-fractured polymer are adhering. Complex rearrangement of the chains connecting the various components has been frequently suggested. However, bond scrambling could enhance the recognition of different surfaces.

**FIGURE 3:**
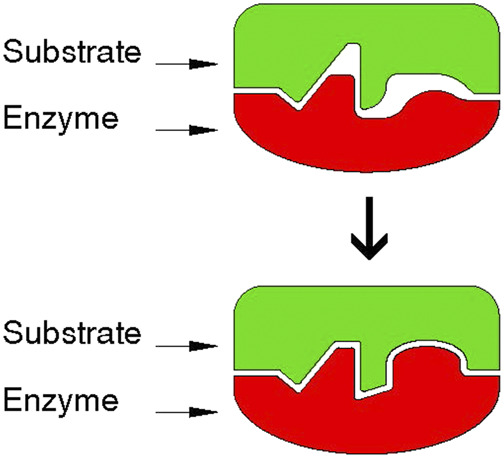
The induced fit in the enzyme–substrate model. Preliminary binding in a limited area is followed by induced synthesis to insure better fit between the surfaces of the enzyme and the substrate, also generating micro interlocking. Adapted from Ciferri 2021. *Designer: Luca Galbusera, 2021.*

A related feature of surface recognition is exhibited by biological systems. In the case of the enzyme-substrate association, new material is synthesized to fill empty cavities occurring between two adhering surfaces (Figure 3) (Ciferri, 2021).

## Concluding remarks

A recent article focuses on theories and simulations related to adhesion, emphasizing problems associated with molecular models and experimental methods for assessing forces and characterizing interfaces at the molecular level (Raos and Zappone, 2021). The quantitative evaluation of van der Waals supramolecular interactions is particularly complicated by the low values of some of the energies involved.

Various authors have therefore avoided direct measurements of the weakest components of the surface energy (*cf.* Eqs 6, 8). The approach suggested here, based on Eq. 5, likewise avoids a detailed evaluation of the van der Waals energy components, but has the advantage of extending to adhesion process concepts and parameters proper to supramolecular chemistry. Wettability might indeed be regarded as the first step in the dissolution of a solvophilic particle in excess solvent. The uncompensated bonds on the external boundary of the particle are saturated by bond exchange with solvent molecules. Dispersive interactions do not prevent the dissolution of the first polymer layer, and the above binding-dissolution sequence is transferred to successive layers on the particle.

It is to be noticed that here is not a clear-cut boundary between the energies represented by χ_12_ and very small binding constants. The identification might be regarded as an operational one, depending upon how small K can be measured.

The present emphasis on the scrambling features of supramolecular polymers highlights a new mechanism that amplifies the local recognition process. Intense motions of chain segments connecting supramolecular groups, or mechanical sliding motions of the surfaces need no longer be postulated. Awaja et al., 2009; Data Physics Instruments, 2021.

## Data Availability

The original contributions presented in the study are included in the article/supplementary material, further inquiries can be directed to the corresponding author.
